# Visualizing Scholarly Trends in Electronic Health (eHealth) Business Models: A Bibliometric Analysis

**DOI:** 10.7759/cureus.71337

**Published:** 2024-10-12

**Authors:** Prageesh C Mathew, Dawn Joseph, Alan Zacharia, Ambili Catherine Thomas, Suby Elizabeth Oommen, Jeena Joseph

**Affiliations:** 1 Commerce, Newman College, Thodupuzha, IND; 2 Economics, St. George's College, Aruvithura, IND; 3 Economics, St. Thomas College, Palai, IND; 4 Commerce, St. Stephen's College, Uzhavoor, IND; 5 Economics, Christian College, Chengannur, IND; 6 Computer Applications, Marian College Kuttikkanam Autonomous, Kuttikkanam, IND

**Keywords:** bibliometric analysis, biblioshiny, business, digital health, e-health, vosviewer

## Abstract

The integration of digital health technologies with innovative business models became a crucial driver in the transformation of healthcare service delivery and management worldwide. As the adoption of electronic health (eHealth) solutions grows, there is increasing scholarly interest in understanding and optimizing these models. This paper reviewed the literature on eHealth business models from 1991 to 2024 through a bibliometric analysis of 1,118 documents published across 711 sources. The analysis primarily focused on journal articles and conference papers, utilizing VOSviewer (Centre for Science and Technology Studies, Leiden University, NLD) and Biblioshiny (Bibliometrix, Naples, ITA) to explore emerging trends, influential authors, and prominent sources. The results revealed a significant rise in research output, especially post-2007, with a peak during the COVID-19 pandemic. Key emerging themes included telemedicine, digital health, and information management, with a strong emphasis on international collaborations, particularly among countries in Europe, North America, and Asia. This study provided valuable insights into the evolution of eHealth business models, highlighting the importance of international and multidisciplinary approaches in this rapidly evolving field.

## Introduction and background

The rapid advancement of digital technologies has profoundly impacted the healthcare industry, driving the emergence and evolution of electronic health (eHealth) business models. These models are essential for ensuring the sustainability and effectiveness of healthcare services, offering innovative approaches to care delivery while addressing concerns related to cost, accessibility, and quality. The increasing digitization of healthcare, including the adoption of telemedicine, wearable health devices, and eHealth records, has necessitated the development of business models that can support these technologies while ensuring alignment with the interests of healthcare providers, patients, and other stakeholders. These eHealth business models aim to balance the demands of a rapidly evolving technological landscape with the need for accessible, high-quality, and affordable healthcare. Moreover, these models are being shaped by regulatory policies, patient expectations, and technological advancements, which continue to influence how healthcare services are structured and delivered. Oderanti and Li developed a commercialization framework for eHealth innovation in the UK healthcare sector. Their model emphasizes the need for sustainability, especially via stakeholder involvement during the design and implementation phases to ensure that the models reflect demands within the market [1]. Along the same line, a case study by van Meeuwen et al. developed a business model regarding online precare services, emphasizing that value cocreation among actors such as health specialists and IT professionals needs to be significant to increase the quality of the offered services [2].

The eHealth system implementation also faces unique challenges, especially in resource-limited settings. Kalayou et al. conducted a study that focused on the applicability of the technology acceptance model in such settings and identified major factors that are thought to influence the adoption of eHealth systems, namely technical infrastructure and user attitude [3]. Qusef et al., in their work, used the HealthGate Cloud (HealthGate Data Corp., Burlington, MA, USA) that focuses mostly on the centralization of patient records and integrates the healthcare providers as one system, hence facilitating ease of sharing data and enhancing the provision of care [4]. Active participation from different stakeholders is required to make eHealth business models effective. In 2015, Limburg et al. updated the importance of the engagement of stakeholders regarding the successful implementation of eHealth-using business modeling as an enabler to guide the interaction among stakeholders and to ensure that this technology best serves the user's needs [5]. This approach is also supported by Vimarlund et al., who explained that broker business models help different groups in the eHealth market, such as healthcare providers and patients, to interact and complete transactions more efficiently. These models are especially useful in social care settings, where different parties need to work together smoothly to deliver care [6].

Advanced technologies like cloud computing and blockchain are increasingly being integrated into eHealth systems to enhance security and efficiency. For example, Hyla and Pejas [7] proposed a blockchain-based integrity model for eHealth systems to address challenges such as unauthorized access and data modification. Similarly, Suraci et al. [8] examined the security implications of 6G technologies in eHealth, highlighting a business-focused approach to security analysis and development trends in digital healthcare environments.

The impact of these eHealth technologies extends beyond security, as they also reshape business strategies and significantly affect healthcare delivery and patient engagement. Eden et al. [9] reviewed the influence of technologies like electronic medical records (EMRs) and clinical decision support systems (CDSS) on hospital practices. They found that while these innovations could improve organizational efficiency, their impact on clinical outcomes was less clear. Likewise, Barello et al. [10] emphasized the need for more holistic eHealth models that fully engage patients in their care, noting that current models only address limited aspects of patient involvement.

Looking ahead, the future of eHealth business models will likely be shaped by both technological advancements and emerging healthcare needs. For instance, Sukkird and Shirahada's [11] study on eHealth service modeling in developing countries shows that mobile technologies may be more effective in addressing the needs of aging populations, particularly in Asia. Furthermore, the pursuit of models integrated into a coordinated discussion by Al-Sharhan et al. stresses the need for constructing holistic frameworks capable of supporting national-level eHealth implementations in Kuwait [12]. Conclusively, the development of eHealth business models lies at the core of the digital transformation of healthcare today. These have to meet various needs of stakeholders, embed advanced technologies securely, and eventually enhance healthcare provision and patient outcomes. Given the dynamic nature of the field, such future research shall aim to refine these models for sustainability and adaptability within an ever-changing healthcare landscape.

The concept and purpose of bibliometric analysis is to provide clarity. Bibliometric analysis is a quantitative method used to systematically assess and analyze scientific literature. It offers valuable insights into the patterns, trends, and influence of scholarly publications [13-17]. This method typically involves examining citation networks, co-authorship relationships, and keyword co-occurrence to understand the structure and evolution of research fields [18-21]. By integrating these tools early in the discussion, readers can better understand how bibliometric analysis shapes a study's approach to exploring eHealth business models. Due to the flexibility and commanding statistical capabilities through integrated development environments like RStudio (Posit, Boston, MA, USA), bibliometric analysis is attracting broad interest among researchers. Biblioshiny (Bibliometrix, Naples, ITA) is integrated as a web application in RStudio. Through its friendly interface, bibliometric analysis can be done independently with a basic knowledge of programming languages. This enables a broad range of analyses, such as citation analysis, cocitation networks, and thematic mapping [22-24]. The VOSviewer (Centre for Science and Technology Studies, Leiden University, NLD) is another specialized tool in bibliometrics, mainly applied to the construction and visualization of bibliometric networks. It is good at mapping and clustering networks of authors, journals, or keywords. The intuitive visual representation lets researchers interpret complex bibliometric data without further complications. These tools together allow an in-depth exploration of academic literature, hence making the discovery of trends and key contributors in a given domain of research easier [25,26].

The major goals of the research are a comprehensive profiling of the scholarly landscape on eHealth business models by detecting key trends, influential authors, and core research themes within the area in question. This study aims to chart the development of research into business models and eHealth from 1991 up to 2024, visualize the rise of publication activity over time, and examine the structural variation of topics. It also seeks to independently determine the collaboration of countries and authors and the distribution of the research output across various academic sources. With VOSviewer and Biblioshiny as tools to conduct the bibliometric analysis, this review provides an in-depth picture of the structure of interlinked research topics and the emphasis on specific keywords in the historical development of key concepts in eHealth business models. Ultimately, the study seeks to provide insights into the current state of research in this area, identifying gaps and opportunities for future explorations.

## Review

Materials and methods

For this study, publications were retrieved using the keywords "E-health," "Ehealth," or “Digital Health” combined with "Business" without applying any language restrictions. The focus was exclusively on journal articles, book chapters, and conference papers. The search yielded a total of 1,118 documents from 711 different sources, spanning from 1991 to 2024. The methodology employed for paper selection in this analysis followed a three-step process. The initial step involved identifying and extracting relevant data from selected databases. In the second step, the data was refined by excluding reviews, editorials, letters, notes, and short surveys, thereby narrowing the focus to articles, book chapters, and conference papers. The refined dataset was then stored as a comma-separated values (CSV) file, and the subsequent bibliometric analysis was conducted using VOSviewer and Biblioshiny software.

The search query utilized in the databases was: (TITLE-ABS-KEY("E-Health") OR TITLE-ABS-KEY("Ehealth") OR TITLE-ABS-KEY("Digital Health") AND TITLE-ABS-KEY(Business)). Further filtering included only articles, book chapters, and conference papers, ensuring a focused analysis of scholarly contributions in this domain. The key findings from the bibliometric analysis, as presented in Table 1, provide a comprehensive overview of the dataset. The time span of the collected documents ranges from 1991 to 2024, with 711 sources yielding a total of 1,118 documents. The annual growth rate of publications was 12.97%, with an average document age of 7.69 years. The documents had an average of 11.72 citations per document, and the total number of references across all documents was 34,187. In terms of document content, there were 5,331 keywords plus (ID) and 3,097 author's keywords (DE) associated with the documents. The authorship analysis revealed a total of 3,654 authors, with 134 of them contributing single-authored documents. The collaboration metrics indicated that there were 142 single-authored documents, with an average of 3.73 co-authors per document and an international co-authorship rate of 22.63%. The document types included 536 articles, 150 book chapters, and 432 conference papers, illustrating the diverse range of scholarly outputs in the field of eHealth business models.

**Table 1:** Important aspects of the study

Description	Results
Main information about the data	
Timespan	1991 to 2024
Sources (journals, books, etc)	711
Documents	1,118
Annual growth rate (%)	12.97
Document's average age	7.69
Average citations per document	11.72
References	34,187
Document contents	
Keywords plus (ID)	5,331
Author's keywords (DE)	3,097
Information on authors	
No. of authors	3,654
Authors of single-authored documents	134
Collaboration	
Single-authored documents	142
Co-authored per document	3.73
International co-authorships (%)	22.63
Document types	
Article	536
Book chapter	150
Conference paper	432

Annual scientific production

**Figure 1:**
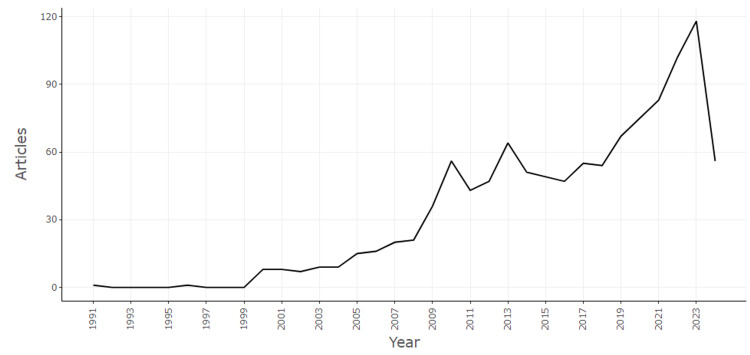
Annual scientific production related to eHealth business models Graph created by the authors.

Figure 1 illustrates the annual scientific production related to eHealth business models from 1991 to 2024. The trend shows a gradual increase in the number of articles published each year, with notable growth beginning around 2007. This growth accelerates significantly after 2011, reflecting a rising interest and expansion in research within this domain. The number of publications peaks in 2022, with over 100 articles published, indicating a heightened focus on eHealth business models, likely driven by the increasing adoption of digital health technologies and their relevance in modern healthcare systems. However, there is a noticeable decline in 2024, which may be attributed to several factors such as changes in research funding, shifts in academic interest, or delays in publication processes. This trend underscores the evolving nature of scholarly attention in eHealth business models, with periods of rapid growth followed by stabilization or decline.

Most relevant authors

**Figure 2:**
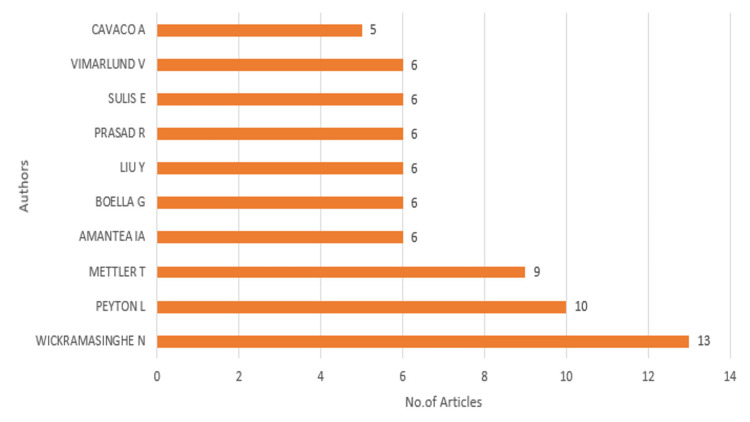
Most relevant authors in the field of eHealth business models Graph created by the authors.

Figure 2 presents the most relevant authors in the field of eHealth business models, ranked by the number of articles they have published. Wickramasinghe N emerges as the most prolific author, with 13 articles, followed by Peyton L, with 10 articles. Mettler T also stands out with nine publications. The remaining authors, including Vimarlund V, Sulis E, Prasad R, Liu Y, Boella G, and Amantea IA, each contributed six articles to this research area, while Cavaco A authored five articles. The distribution suggests that a few key researchers, notably Wickramasinghe N and Peyton L, have made substantial contributions to the scholarly discourse on eHealth business models, possibly indicating their leading roles in this research domain. The presence of multiple authors with a significant number of publications highlights a robust and active research community focused on advancing knowledge in eHealth business models. This concentration of contributions may also suggest collaborative networks and repeated efforts by these researchers to explore different aspects of eHealth business models over time.

Most relevant sources

**Figure 3:**
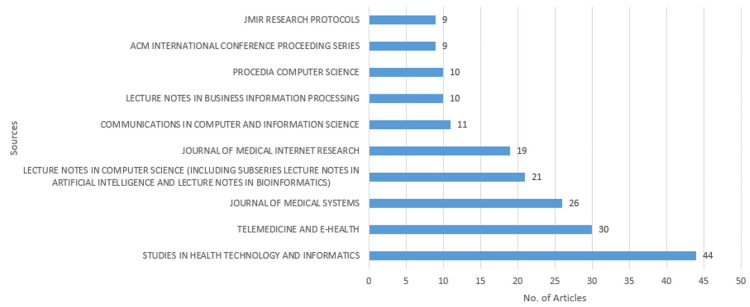
Most relevant sources concerning eHealth business models JMIR: Journal of Medical Internet Research, ACM: Association for Computing Machinery Graph created by the authors.

Figure 3 highlights the most relevant sources for publications related to eHealth business models. *The Studies in Health Technology and Informatics* stands out as the leading source, with 44 articles published, indicating its significant role in disseminating research in this field. Telemedicine and eHealth follow with 30 articles, showcasing their importance in the intersection of digital health and business models. Other key sources include the *Journal of Medical Systems* with 26 articles and the *Lecture Notes in Computer Science* (including a subseries in artificial intelligence and bioinformatics), which has published 21 articles. The *Journal of Medical Internet Research* also plays a critical role with 19 articles, reflecting its focus on internet-based health research. Lesser but still notable contributions come from sources such as *Communications in Computer and Information Science* (11 articles), *Lecture Notes in Business Information Processing* and *Procedia Computer Science* (10 articles each), and *JMIR Research Protocols* and the *ACM International Conference Proceeding Series *with nine articles each. These sources collectively represent a diverse array of journals and conference proceedings that contribute to the academic conversation on eHealth business models, indicating a multidisciplinary approach that spans health technology, medical systems, computer science, and informatics.

Thematic map

**Figure 4:**
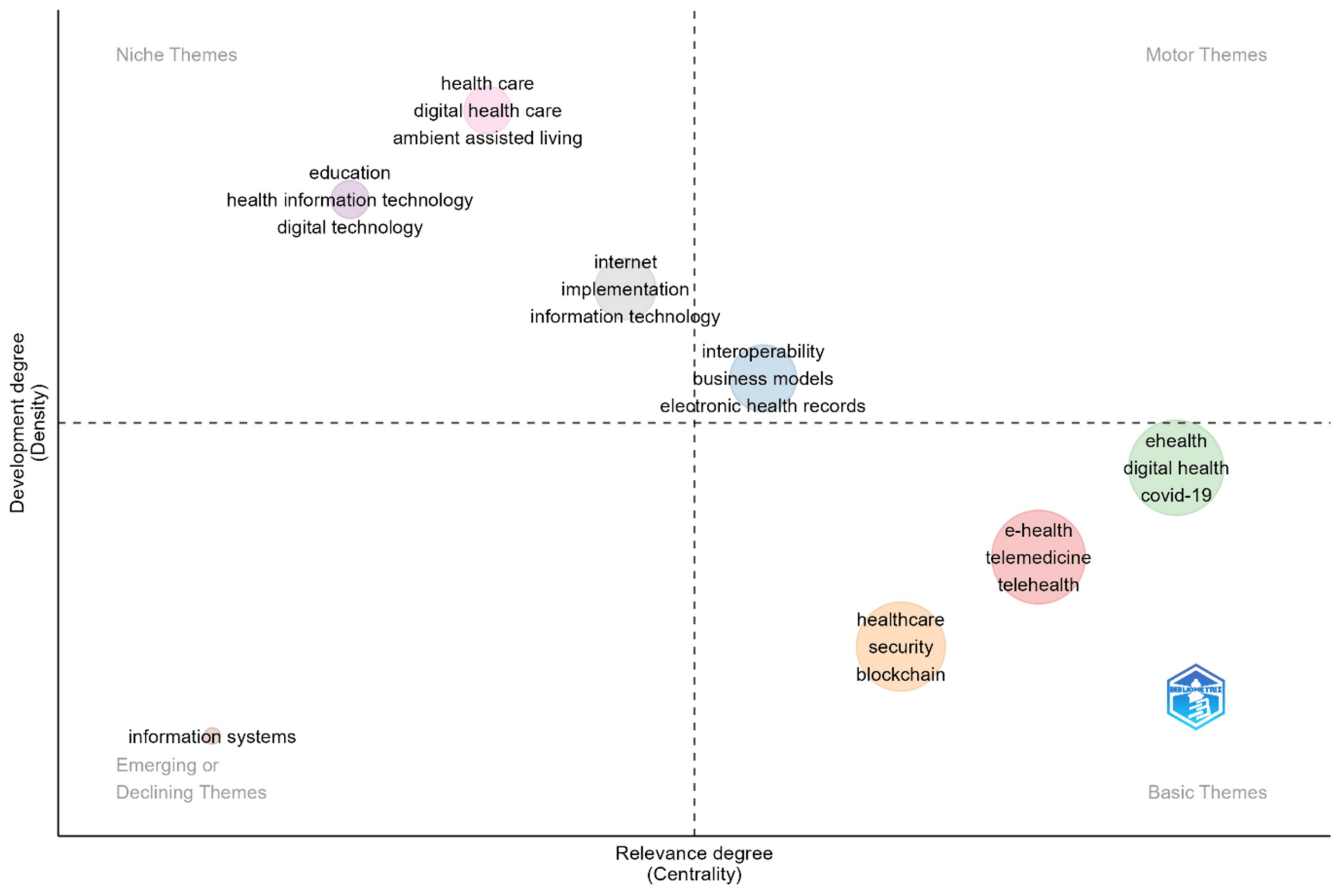
Thematic map categorizing various themes related to eHealth business models Diagram created by the authors.

Figure 4 provides a thematic map that categorizes various themes related to eHealth business models based on their degree of development (density) and relevance (centrality). The map divides the themes into four quadrants. Motor themes are located in the upper right quadrant, representing well-developed and highly relevant areas of research, such as "eHealth," "digital health," and "COVID-19," which have become central to the field, particularly during and after the pandemic. Niche themes, found in the upper left quadrant, are highly developed but less central, indicating specialized research areas like "health care," "digital health care," and "health information technology." These themes are well-established but more specific to certain subfields within eHealth business models. Basic themes in the lower right quadrant are central but less developed, encompassing foundational concepts such as "e-health," "telemedicine," and "business models," which are essential to the field but require further exploration. Finally, the lower left quadrant contains emerging or declining themes, which are less developed and less central, potentially indicating either emerging trends or areas of research that are losing relevance, such as "information systems." This thematic map offers a comprehensive overview of key areas of focus within eHealth business models, highlighting both well-established and emerging topics in the field.

Trend topics

**Figure 5:**
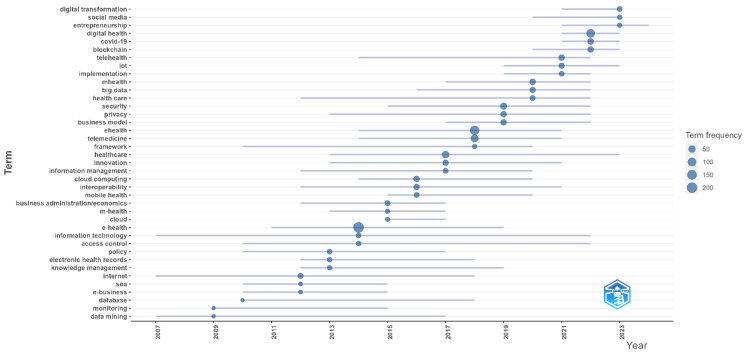
Evolution of trend topics in eHealth business models Figure created by the authors.

Figure 5 shows the evolution of trend topics in eHealth business models over time. Terms are plotted against their frequency of appearance from 2007 to 2023. The size of the bubbles is representative of the term frequency, i.e., the larger the bubble, the more the more indicative of high frequency. This shows quite clearly the evolution of topics, with more general foundational concepts, such as "e-business," "cloud computing," and "information technology," dominating the earlier years. As time progresses, other more fine-grained and emergent topics, such as "big data," "blockchain," "digital health," and "COVID-19," come to the fore, reflecting how the field adapts to technological developments and global events. This trend analysis underlines how terms related to "digital transformation," "telehealth," and "COVID-19" have grown in importance, especially within the last couple of years, but seem to shift toward more contemporary and relevant issues in eHealth. Other terms, such as "security," "privacy," and "interoperability," appear throughout the timeline continuously and thus seem to remain relevant with regard to discussions on eHealth business models. It clearly shows the evolution of the focus in this area over time, with a striking shift toward digital and data-driven health solutions in more recent years.

Three-field plot

**Figure 6:**
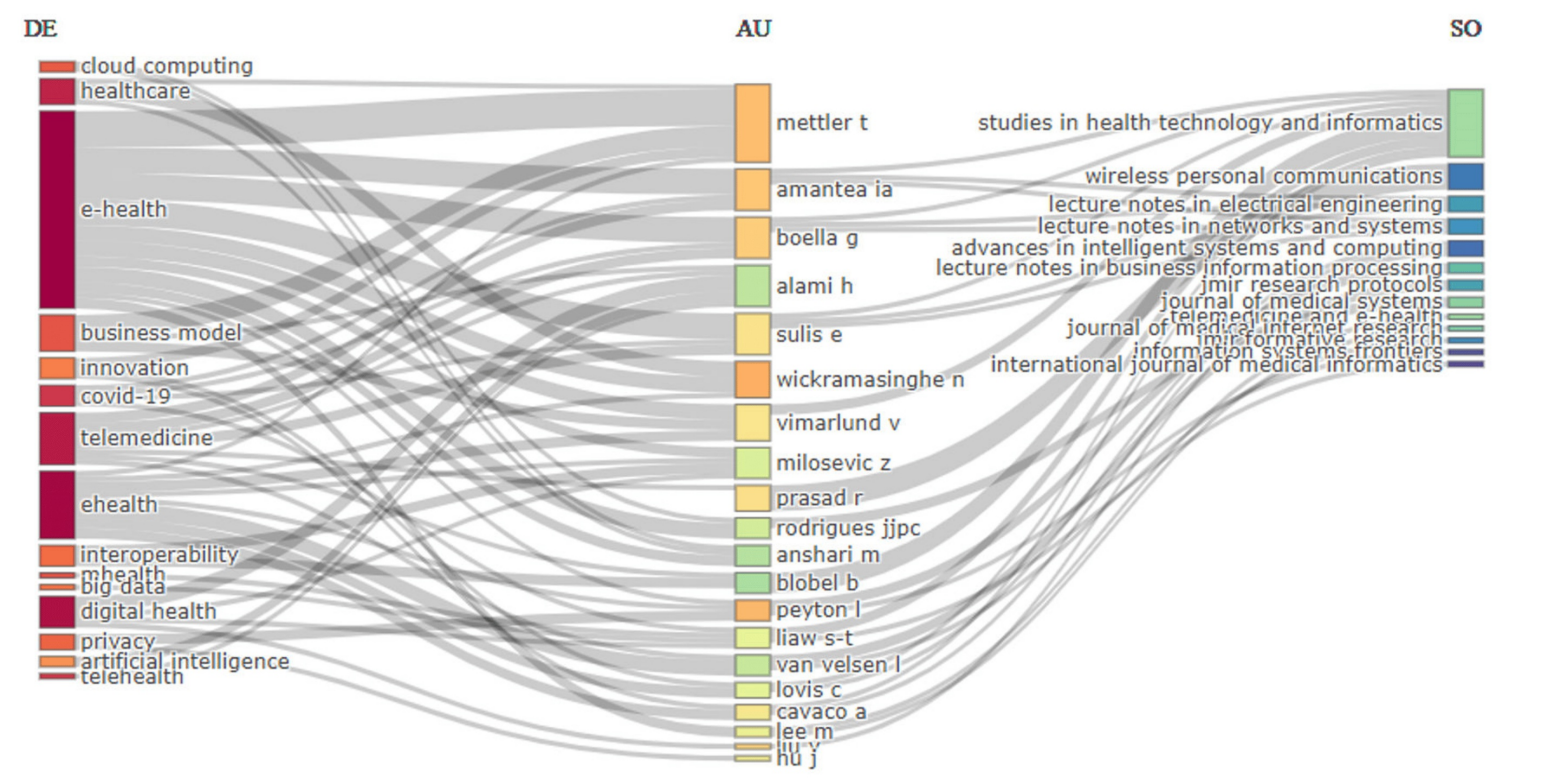
A three-field plot illustrating the relationships between key research keywords (left), contributing authors (center), and publication sources (right) in the field of rHealth business models. The plot highlights the primary areas of focus, active researchers, and the sources where significant work is published, providing a comprehensive view of the research landscape. Diagram created by the authors.

Figure 6 shows a three-field plot, in which, from left to right, the given keywords, authors, and sources are visualized, giving an overview of the relationship between highly used keywords, authors who contributed, and their journal or source of their publication. Excluding general words, the keywords featured on the left were "e-health," "business model," "innovation," and "telemedicine," thus showing the areas where the key focus usually lies in eHealth business models. Authors feature in the central field; among the heavy contributors are Mettler T, Amantea IA, Boella G, and Sulis E. The lines connecting these authors to the keywords show the topics they are most associated with, highlighting their research interests. On the right are the sources or journals these authors publish in. The most central sources are *Studies in Health Technology and Informatics*, *Journal of Medical Internet Research*, and a few *Lecture Notes* series. The links between the authors and these sources show via which sources the highly influential research is being published. This three-field plot shows how specific authors are associated with particular research themes and how the topics in turn are reflected in various academic journals. It shows the landscape of research in eHealth business models and the interplay among the topics, investigators, and publication venues.

Co-authorship between countries

**Figure 7:**
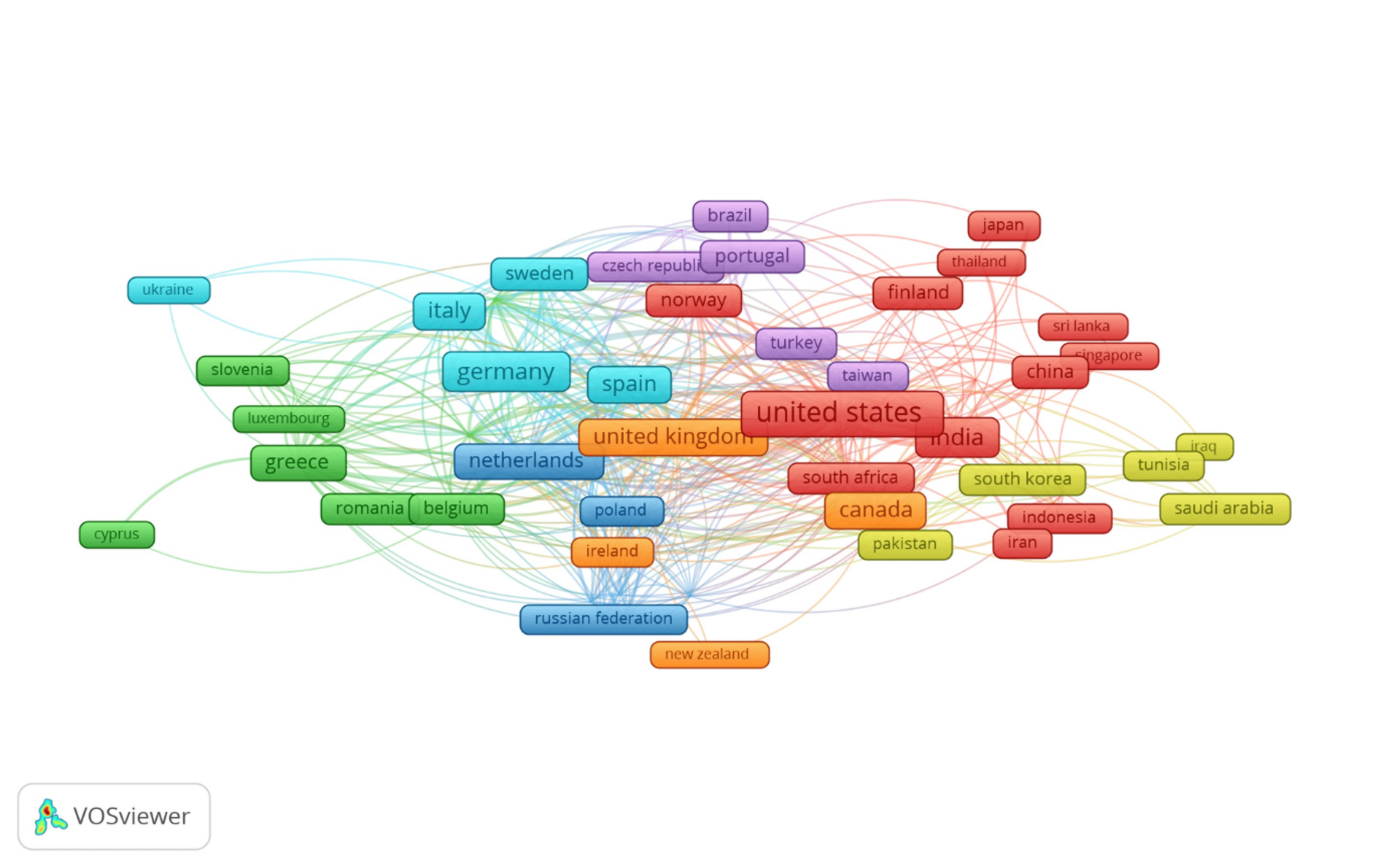
Co-authorship between countries highlighting the global nature of research into eHealth business models Diagram created by the authors.

Figure 7 presents a co-authorship network map that illustrates the collaborative relationships between countries in the field of eHealth business models. Each node on this map represents a country, while nodes are sized by volume. Lines connect nodes based on their co-authorship links, therefore highlighting the intensity of collaboration between them. The map of the densely interconnected network of research across the world shows that some countries are hubs, which quite nicely capture international collaboration. The data behind this map show color-coded clusters, representing different groups of countries with strong co-author connections. Surprisingly, the United States, India, Canada, China, and a few other Asian and North American countries come to make the cluster red, showing these countries as the leading contributors to collaborative research in the world. It is especially the United States that forms a big co-authorship center, connecting with a wide array of countries across different clusters, reflecting its leading role in the research community and its extensive international partnerships.

The green cluster includes countries like Greece, Italy, Germany, and the Netherlands. This would suggest that these European countries, by appearance, hold a strong collaborative relationship with their counterparts, which underlines the regional dimension of their research collaborations. Within this cluster, both Germany and Italy emerge as the important actors with multiple co-authorships links to the other European countries beyond links to countries outside the cluster, denoting their influential position in the eHealth-research domain. The blue cluster is comprised of the UK, Spain, Sweden, and the Netherlands. Here, the UK maintains a major connector position. Accordingly, the strong connections between these countries reflect a well-developed network of European research collaborations. The U Kthus plays a significant role in connecting different parts of the global research community.

The other marked clusters include the purple cluster, constituted by countries such as Brazil, Portugal, and Norway, suggesting active collaborations among European and South American countries, primarily. The yellow cluster, in which countries like South Africa, Saudi Arabia, and Iran are connected, is indicative of collaborations beyond Europe and North America. This co-authorship map underlines the global nature of research into eHealth business models, with many countries making a contribution and collaborating with others on this important topic. Distinct clusters reflect regional concentrations of research activity, and the extensive connections between clusters reflect the cross-regional collaborations driving innovation and knowledge sharing in this field. The visualization emphasizes international cooperation in fostering research and implementation of eHealth business models.

Co-occurrence of keywords

**Figure 8:**
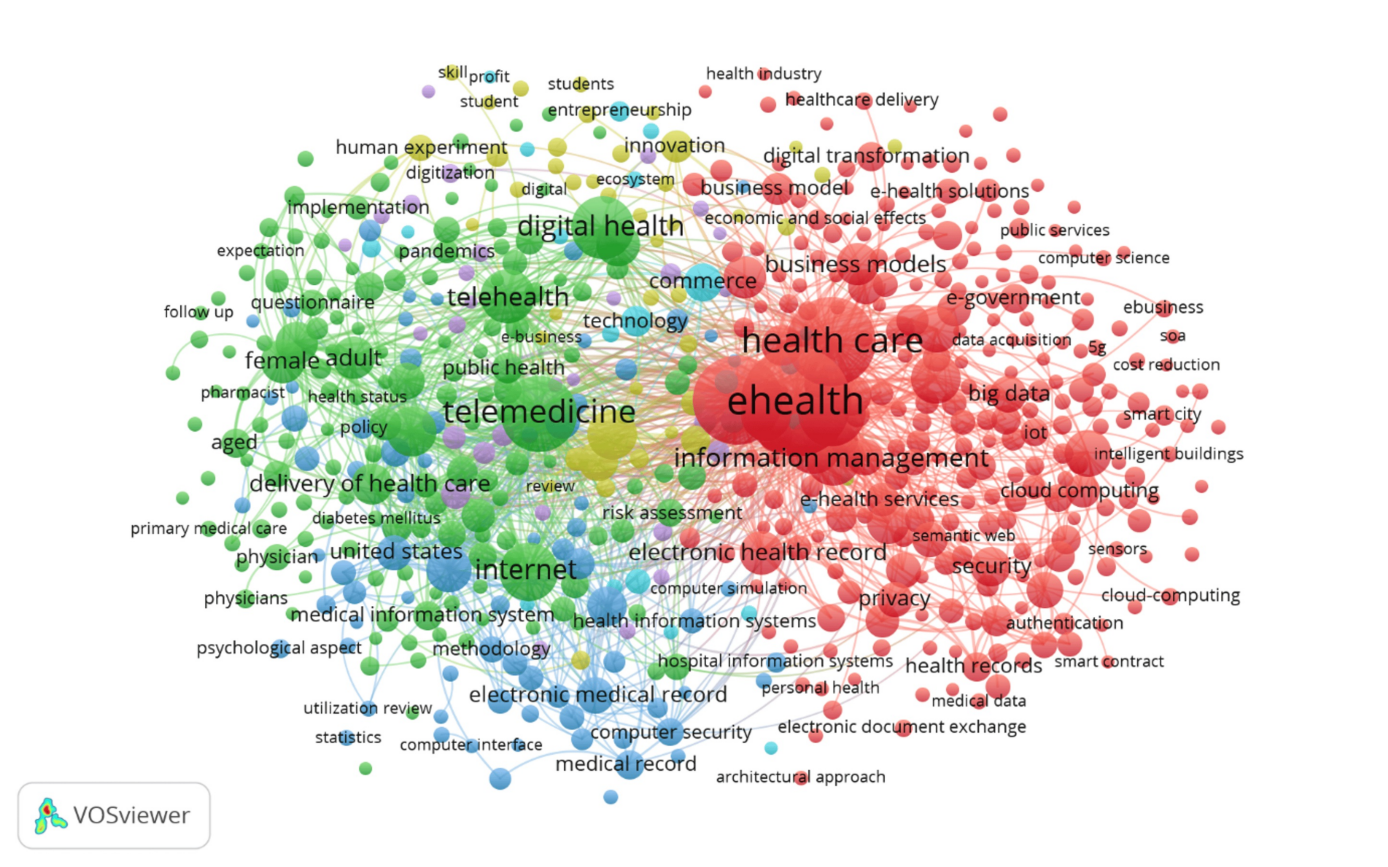
Co-occurrence of keywords in the field of eHealth business models Diagram created by the authors.

Figure 8 presents a co-occurrence network map of keywords in the field of eHealth business models, visualizing the relationships and frequency of keywords that appear together in the literature. Each node represents a keyword, with the size of the node indicating the frequency of its occurrence. The lines connecting the nodes represent the co-occurrence of keywords within the same documents, illustrating the interconnectedness of various research topics. The map is color-coded into clusters, each representing a group of related keywords that frequently appear together, highlighting different thematic areas within the field. The red cluster is the most prominent, centered around the keywords "eHealth," "health care," and "information management." This cluster reflects the core focus of research on the integration of digital technologies into healthcare management and delivery, including topics like "business models," "cloud computing," and "privacy." The size and centrality of the nodes within this cluster indicate that these topics are foundational to the field of eHealth business models and are frequently studied together. The green cluster focuses on keywords related to "telemedicine," "telehealth," and "digital health." This cluster emphasizes the growing importance of remote health services and digital platforms in healthcare, with related terms such as "pandemic," "public health," and "delivery of health care." The presence of these terms highlights the impact of global health crises, such as the COVID-19 pandemic, on accelerating the adoption and study of telehealth technologies.

The blue cluster is centered around keywords such as "internet," "electronic medical record," and "electronic health record," reflecting the technological infrastructure that underpins digital health initiatives. This cluster also includes terms like "interoperability" and "security," pointing to the technical challenges and considerations in ensuring effective and secure data exchange across healthcare systems. The yellow cluster appears to focus on innovation and emerging technologies, with keywords like "digital transformation" and "technology." This cluster suggests a forward-looking perspective within the field, exploring how new technologies can drive changes in healthcare delivery and business models. Overall, this co-occurrence map provides a detailed overview of the major research themes in eHealth business models, illustrating how different topics are interrelated and identifying key areas of focus within the literature. The clusters reveal the multidisciplinary nature of the field, integrating aspects of healthcare, technology, business, and public health, and underscore the importance of certain themes that are central to ongoing research and development in eHealth.

Reference publication year spectroscopy (RPYS) of eHealth business models research

**Figure 9:**
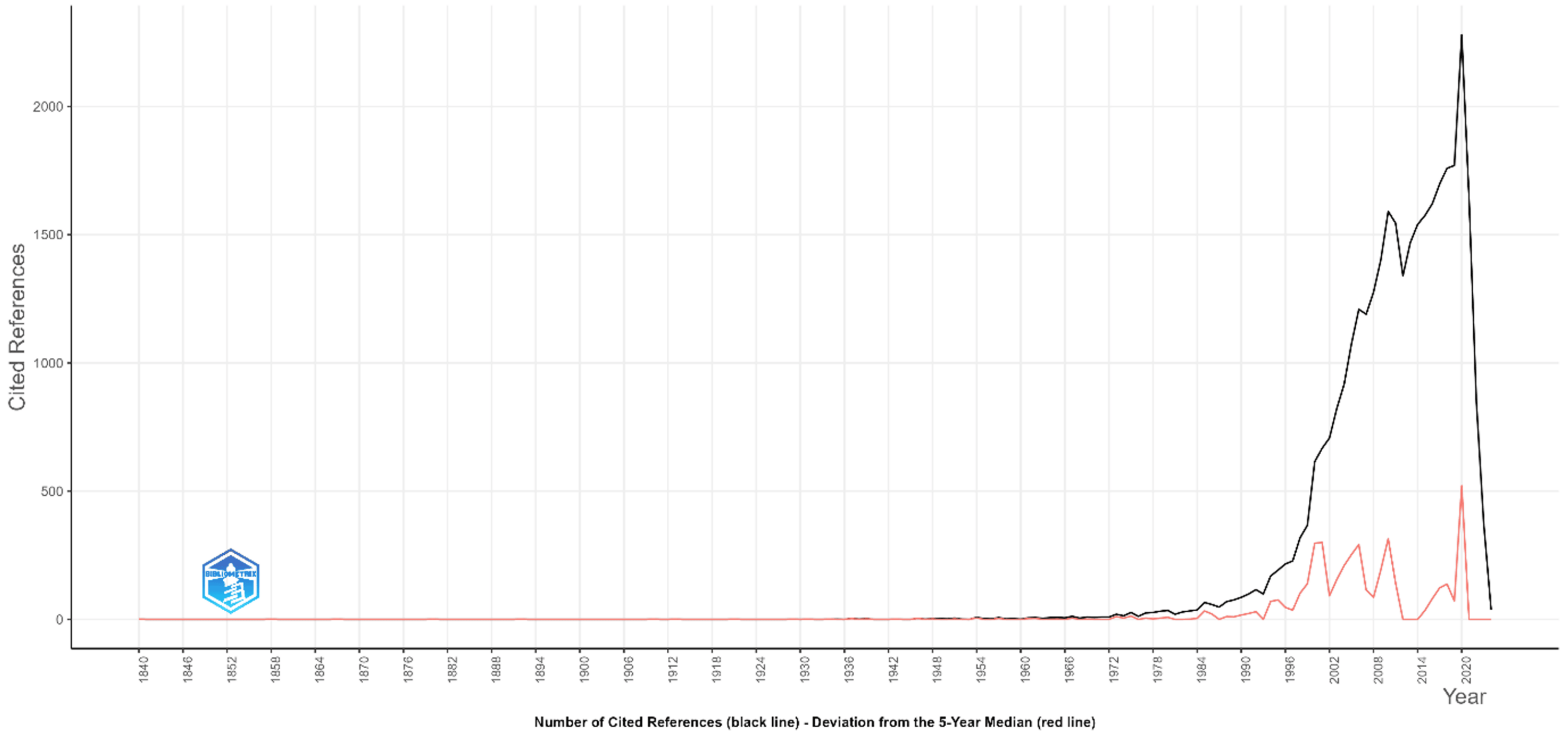
An RPYS graph showing the number of cited references over time in the field of eHealth business models. RPYS: Reference publication year spectroscopy Graph created by the authors.

Figure 9 presents an RPYS graph that tracks the number of cited references over time in the field of eHealth business models. The black line represents the total number of cited references each year, while the red line indicates deviations from the five-year median, highlighting periods of increased citation activity that may correspond to significant developments or publications in the field. The graph shows a marked increase in the number of cited references starting around the 1980s, with a sharp rise beginning in the early 2000s. This trend likely reflects the growing importance and rapid development of digital health technologies, telemedicine, and eHealth business models during this period. The peak in cited references around 2020 suggests a particularly active period for foundational research, likely influenced by the COVID-19 pandemic, which significantly accelerated the adoption of digital health solutions and led to a surge in related academic publications. The red line's deviations indicate specific years where the number of cited references significantly deviates from the five-year median, pointing to potential landmark studies or key publications that have had a lasting impact on the field. This pattern of citation activity provides insights into the historical development of research in eHealth business models, showing how certain periods have contributed more heavily to the foundational knowledge and ongoing discourse in this area. The sharp decline after 2020 could be due to the natural lag in citation accumulation for more recent publications or a reflection of a peak in pandemic-related research that has since stabilized.

Discussion

The last two decades have seen a significant change in the business model landscape of eHealth. This is reflected both in the increased number of scholarly publications and in thematic development areas. Based on the bibliometric analysis conducted, the recognition of digital health as a transforming agent in health delivery has gained greater momentum in recent times; this development was especially triggered by global challenges such as the COVID-19 pandemic. It became evident from this data that key themes of telemedicine, digital health, and business models have become core to the academic discourse, reflecting their critical importance in the ongoing shift toward more integrated technology-driven health systems.

The network of co-authorships underlines the global nature of research in this domain, with strong collaborations among countries like the United States, India, and several European nations. These international collaborations have been illustrative in furthering the field by facilitating cross-pollination of ideas and sharing of best practices. On the other hand, this analysis depicts inequality in research activity, especially in low- and middle-income countries (LMICs), indicating that future research should be even more inclusive in order to also pay attention to the specific challenges faced by areas in such conditions.

Further evidence for the multidimensionality of research into eHealth has been provided by the thematic map, with various clusters pointing toward the convergence of healthcare, technology, and business strategy. It is this convergence that makes the need for developing sustainable and scalable eHealth solutions to adapt to the diverging needs of different healthcare systems ripe. The prominence given to security, privacy, and interoperability themes underlines many of the concerns put forward in relation to ethical and technical challenges in deploying the swath of digital health technologies at scale.

Research Gaps and Future Directions

In spite of the remarkable volume of studies concerning business models in eHealth, several important gaps persist and provide opportunities for future research. First of all, there is a remarkable lack of longitudinal studies focused on how such models can be viable and then scale up over time in different care settings. While much of the existing research currently focuses on either short-term outcomes or specific case studies, there is a need for more comprehensive analyses tracking how eHealth business models evolve over time and how they can be taken to scale.

While artificial intelligence and blockchain for this matter are emerging technologies most echoed in the literature, the empirical studies that show their actual implementations, effectiveness, and challenges are few. Further studies should be conducted in exploring such arenas to provide insights on how such technologies might be leveraged to further eHealth efficiency and security.

Another area needing deliberation is ethics, especially on data privacy and consent of patients. The understanding of these ethical issues and their addressability is bound to become increasingly essential as the solutions for digital health start to mushroom across the world, more so in LMICs where even regulatory frameworks may not be firm enough. Further, comparative studies of how cultural, socio-economic, and regulatory factors in different regions drive the success of eHealth business models could help develop models that are adaptable for various environments.

Lastly, it is recommended that more studies be conducted on eHealth engagement and user experience from the perspective of the patients. It is from this insight into how patients interact with digital health platforms, experience challenges, and the elements that contribute to successful engagement that one can enable solutions that are easily usable and accessible to achieve better healthcare outcomes.

Practical Implications

The practical implications of these findings are threefold for stakeholders in the health sector. For healthcare providers, the centrality of telemedicine and digital health within the research literature underlines a need for investment in infrastructure and training that support these services effectively. It is worth considering for providers to adopt flexible business models that can adapt to the quick pace of technological change and evolving patient expectations.

To the policymakers, it points to the international dispersion of research activity and emphasizes international collaboration, further developing harmonized regulatory frameworks that enable cross-border research and implementation. Finally, policymakers should create an enabling environment for the adoption of digital health technologies, regulatory barriers, data privacy, and security, and support innovation through target funding, incentives, and other measures.

Therefore, technology developers and entrepreneurs would realize, in consideration of digital health, telemedicine, and business model research, that there is a demand for scalable, secure, and user-friendly digital health solutions. Developers should include user-centered design, interoperability, and ethical considerations in the course of product development. Each of the above-listed focus areas has the potential to help stakeholders contribute to developing effective, sustainable, and equitable eHealth solutions that improve healthcare delivery and outcomes globally.

## Conclusions

Results from the bibliometric analysis performed in this study reflect the nature of research into eHealth business models as dynamic and in rapid evolution. The disproportional growth in publications over the last decade will give, in addition, much relevance to the field of digital health technologies and respective business models within modern healthcare systems. Key themes discussed here, which have emerged throughout the years as central areas of focus, concerns that are driven both by technological advances and global health crises like the COVID-19 pandemic, include telemedicine, digital health, and information management. The study also describes a very collaborative global research network with significant contributions from countries across Europe, North America, and Asia. This international cooperation has been pivotal in driving innovation and knowledge sharing in eHealth business models. This signals that future research in the field, as the field continues to evolve, should sustain the search into the integration of other emerging technologies, such as artificial intelligence and blockchain while responding to various challenges around privacy, security, and interoperability. The conclusions derived from this analysis may help policymakers, researchers, and practitioners work toward developing sustainable and efficient eHealth business models in the years ahead.
